# Improving prognostication of pneumonia among elderly patients: usefulness of suPAR

**DOI:** 10.1186/s12877-024-05270-0

**Published:** 2024-08-24

**Authors:** Artida Ulaj, Arni Ibsen, Leire Azurmendi, Jean-Charles Sanchez, Virginie Prendki, Xavier Roux

**Affiliations:** 1 Division of Anesthesiology, Department of Acute Medicine, Geneva University Hospital, Geneva, Switzerland; 2 Department of Internal Medicine Specialties, Medical Faculty, Geneva University Hospitals, Geneva, Switzerland; 3 Medical Faculty, Geneva, Switzerland; 4 Division of Infectious Diseases, Department of Internal Medicine, Geneva University Hospital, Geneva, Switzerland; 5 Division of Geriatric Medicine, Department of Rehabilitation and Geriatrics Medicine, Geneva University Hospitals, Thônex, Switzerland; 6 Division of Intensive Care, Department of Acute Medicine Geneva, Geneva University Hospital, Geneva, Switzerland

**Keywords:** Geriatric, Pneumonia, Biomarker, Mortality

## Abstract

**Purpose:**

Elderly patients with suspected pneumonia represent a significant proportion of hospital admissions, which is a prognostic challenge for physicians. Our research aimed to assess the prognosis of patients with pneumonia using soluble urokinase plasminogen activator receptor (suPAR) combined with clinical data.

**Methods:**

In a prospective observational study including 164 patients > 65 years (mean age 84.2 (+/-7.64) years) who were hospitalized for a suspicion of pneumonia, suPAR was assessed for each patient, as was the prognosis score (PSI, CURB65) and inflammatory biomarkers (C-reactive protein, procalcitonin, white blood cells). The prognostic value of the suPAR for 30-day mortality was assessed using receiver operating characteristic (ROC) curve analyses. Optimal cut-offs with corresponding sensitivity (SE) and specificity (SP) were determined using the Youden index.

**Results:**

A suPAR > 5.1 ng/mL was predictive of 30-day mortality with a sensitivity of 100% and a specificity of 40.4%. A combination of the following parameters exhibited an SE of 100% (95% CI, 100–100) for an SP value of 64.9% (95% CI, 57.6–72.2) when at least two of them were above or below the following cut-off threshold values: suPAR > 9.8 ng/mL, BMI < 29.3 kg/m2 and PSI > 106.5.

**Conclusion:**

The suPAR seems to be a promising biomarker that can be combined with the PSI and BMI to improve the prognosis of pneumonia among elderly patients. Prospective studies with larger populations are needed to confirm whether this new approach can improve patient outcomes.

**Trial registration:**

ClinicalTrials.gov (NCT02467192), 27th may 2015.

**Supplementary Information:**

The online version contains supplementary material available at 10.1186/s12877-024-05270-0.

## Key summary points

The prognosis of pneumonia in geriatric patients remain challenging, can the dosage of suPAR help clinicians determine the prognosis of pneumonia among geriatric patients? The use of individual or clinical parameters should be improved for a better prediction of respiratory complications and mortality. Adding suPAR dosage with PSI and BMI provides the best sensitivity and specificity for 30-day mortality.

## Introduction

Pneumonia is a major cause of death and a frequent cause of hospitalization, mostly affecting the geriatric population [1]. The annual incidence of community-acquired pneumonia (CAP) for noninstitutionalized patients is estimated to be 18.2 cases per 1000 persons in persons aged 65–69 years and 52.3 cases per 1000 persons in those aged 85 years or older [2]. Mortality is also considerably greater in elderly patients. The prognostic assessment tools usually used are the pneumonia severity index (PSI) and CURB-65, as well as routine inflammatory biomarkers such as C-reactive protein (CRP) and procalcitonin (PCT). The PSI classifies patients into 5 classes according to the expected one-month mortality [3]. The CURB-65 score is also used but has a lower performance [4]. However, these scores have a low sensitivity to detect patients with worse outcomes, especially among elderly patients [5]. The adjunct of biomarkers such as CRP and PCT with clinical scores (such as PSI) (has previously been studied and improved [6]. Nevertheless, the specificity of CRP and ProCT remain discussed [7].

The soluble urokinase plasminogen activator receptor (suPAR) is a nonspecific inflammatory biomarker with promising results as a predictive marker of mortality (28-D) in sepsis, critical illness and COVID-19 [8–10].

It has previously been shown that the suPAR has a superior prognostic performance to that of CRP or PCT and is associated with a greater risk of death in patients with systemic inflammation [11, 12]. Improving the predictive scores of pneumonia complications among elderly patients could lead to better patient orientation, reduced hospitalization costs and improved outcomes [13]. We aimed to assess whether the SuPAR could improve the prognostic assessment of pneumonia among elderly patients when it was added to a severity score frequently used in the emergency department (ED), such as the PSI. To assess the combination of clinical parameters and biomarkers, the Panelomix, a computational toolbox, was used. This method combines biomarkers and clinical scores by selecting thresholds that provide optimal classification performance (description below). We hypothesise that the combined use of PSI with suPAR can provide a more comprehensive and accurate assessment of pneumonia severity and possible clinical outcomes.

## Materials and methods

### Study population

We retrospectively analysed data from a prospective observational cohort of 200 patients treated at Geneva University Hospitals, Switzerland, including geriatric patients with a clinical suspicion of pneumonia based on respiratory and infectious symptoms. The study included consecutive patients over 65 years old who were hospitalized at Geneva University Hospitals between May 1, 2015, and April 30, 2016, with suspected pneumonia based on suggestive signs and symptoms. The diagnosis of pneumonia was determined *a posteriori* by a panel of senior physicians who had access to all clinical, biological, radiological, and microbiological data [14]. Low-dose chest computed tomography (CT) scans were performed on all patients upon admission. Patients who had been treated for pneumonia in the previous 6 months or who had been admitted to the intensive care unit were excluded. Our study focuses on patient prognosis in the context of ED consultations for hospital admission. In our study, this biomarker would not influence the decision of admission in ICU. Patients immediately requiring mechanical ventilation or aminergic support were immediately admitted to intensive care, regardless of the results of clinical scores or biological markers.

The treatment was not modified for the aim of the study and was prescribed at the discretion of the clinician, following local and national guidelines used at the time of recruitment.

The study was approved by the Institutional Review Board of Geneva University Hospitals (CER-14-250) and registered at ClinicalTrials.gov (NCT02467192) on the 27th may 2015, and informed consent was obtained from all patients or their next of kin.

### Data collection

The data, including demographic data, clinical data (including BMI), clinical prognosis score (PSI and CURB 65) and biological data, were prospectively collected by the study nurse.

### Marker measurement

Blood samples were obtained for routine analysis within 48 h of admission to the acute care setting, and one tube was stored in a biobank for later added dosages. We analysed different panels for the best sensitivity, specificity, and positive and negative predictive values for four inflammatory biomarkers: suPAR, CRP, PCT and white blood cell (WBC) count. The plasma concentrations of CRP were measured promptly using immunoturbidimetry. PCT concentrations were determined using a rapid assay with a sensitivity of 0.06 µg/L (Kryptor PCT, Brahms, Hennigsdorf). The plasma suPAR level was measured with a suPARnostic ELISA (ViroGates A/S, Birkerød, Denmark) wich presents a LOD of 0.4ng/mL.

### Outcome

The main outcome was 30-day mortality. The date of death was recorded during hospitalization or obtained by consulting the institutional database and the cantonal register of deaths after discharge.

### Statistical data analysis

The statistical analyses were conducted using SPSS software (version 21, SPSS Inc., Chicago, IL). Since the levels of the different analytes did not follow a normal distribution, the Mann‒Whitney U test was used to compare two unpaired groups. To evaluate whether there were significant differences between patients with favourable and poor outcomes, Fisher’s exact test and the chi-squared test were used.

All the statistical tests were two-tailed, and a p value < 0.05 was considered to indicate statistical significance. At the time of admission to the hospital, receiver operating characteristic (ROC) curves were calculated for suPAR, CRP, PCT, and WBC, as well as for clinical parameters such as age, CURB-65, PSI, and BMI. For each marker, the sensitivity (SE) value was limited to 95–100%, and the cut-off value was selected to avoid false negative results. The pROC package for S+ (version 8.1., TIBCO Software Inc.) was used to determine the areas under the partial area under the curve (AUC), specificity (SP), SE, and 95% confidence intervals (95% CI) [15].

### Panel development

To select the panel, we utilized the PanelomiX tool as described previously [16]. PanelomiX is a computational tool used for generating panels of biomarkers based on predefined thresholds. We obtained optimized cut-off values using modified iterative permutation-response calculations, known as rule-induction-like (RIL) calculations. These calculations included all individual parameters, various analytes (suPAR, CRP, PCT, WBC), and clinical parameters (age, CURB-65, PSI, BMI).

This approach evaluates how each variable contributes to the model in combination with others, adjusting thresholds to select variables that provide unique information while minimizing redundancy caused by multicollinearity. Additionally, we employed random forest analysis to assess the importance of each variable and select only those that significantly contribute to the final model.

The cut-off values of each biomarker or clinical parameter were iteratively adjusted by 2% increment quantiles. This involved selecting thresholds for each biomarker and assessing the robustness of the constructed panels using receiver operating characteristic (ROC) curves and 10-fold cross-validation.

Cross-validation was utilized to assess the stability of selected variables, evaluating model performance on different data subsets to identify consistent and robust variables across various samples, thereby reducing the impact of multicollinearity. Following cross-validation, we compared the obtained ROC curves with those of individual biomarkers using De Long’s method. After each iteration, the specificity (SP) value was calculated with a sensitivity (SE) value set between 95% and 100%. The panel size was limited to a maximum of three parameters. In predicting one-month mortality, our emphasis on high sensitivity aims to minimize false negatives.

## Results

**Fig. 1:**
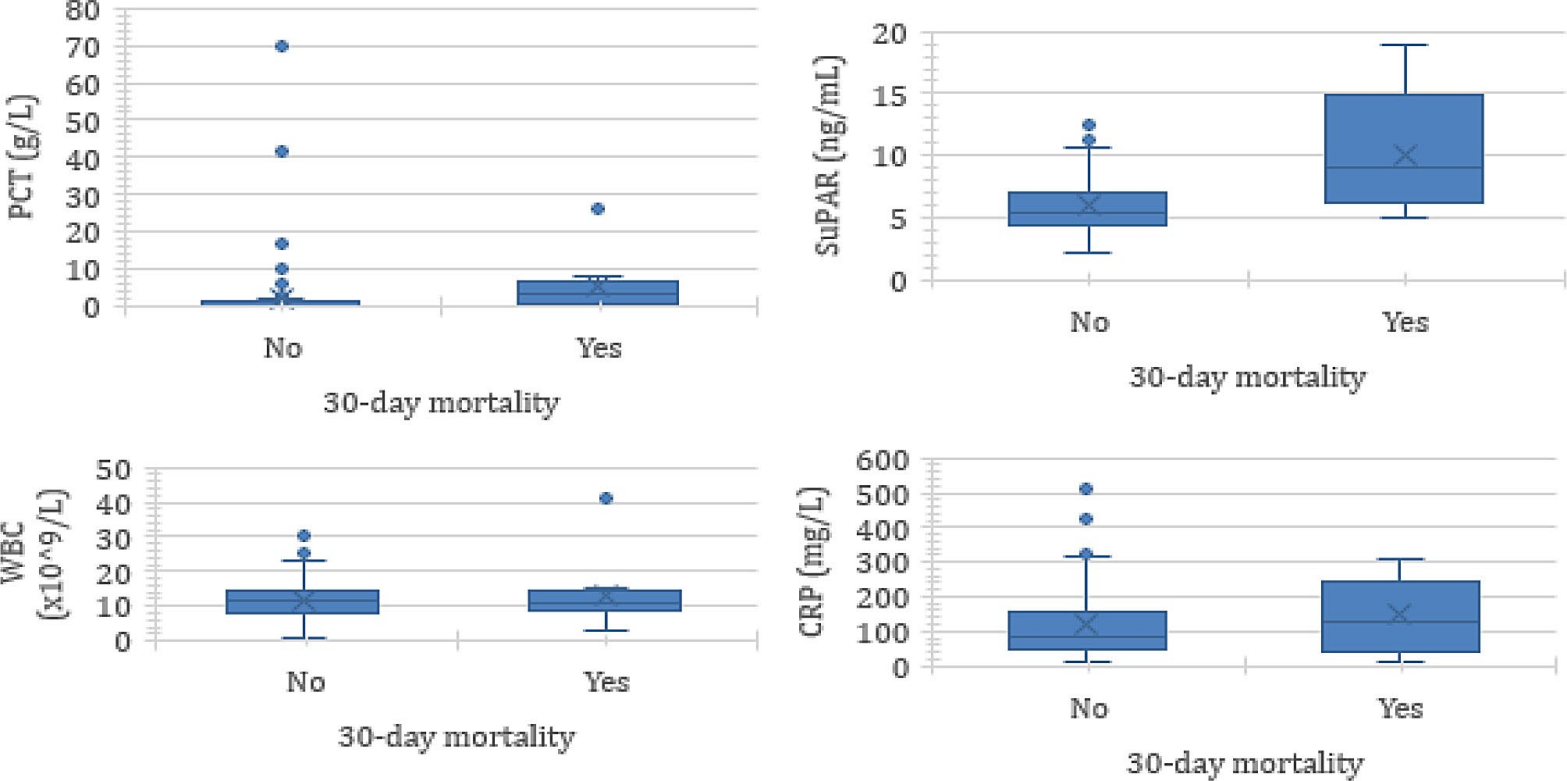
Boxplots showing the values of suPAR, PCT, CRP and WBC according to 30-day mortality, PCT: procalcitonin, BMI: body mass index, CRP: C-reactive protein, WBC: white blood cell count, PSI: pneumonia severity index

Out of the 200 patients in the cohort, one hundred and sixty-four were included, and the exclusion of 36 patients was due to unavailable frozen biological samples for the suPAR dosage. Table 1 displays their demographic, clinical and biological characteristics. The mean age was 84.2 (± 7.6) years, and the majority were male (55.0%). The comorbidities observed among the patients included heart failure (34.8%), diabetes (21.3%), chronic obstructive pulmonary disease (18.3%), dementia (34.8%), and chronic renal failure (23.2%). Within one month, 6% of the patients died. The PSI was significantly greater in patients who experienced one-month mortality than in those who did not die. The average BMI was lower in the one-month deceased patient group than in the surviving patient group (22.4 vs. 25.2 kg/m^2^), but the difference was not statistically significant. Regarding biomarkers, only the suPAR and PCT were associated with 30-day mortality. The concentrations of CRP and WBC were not different. Figure 1 shows the value of biomarkers according to 30-day mortality. PCT and suPAR were greater in patients who died within 30 days.

**Table 1:** Demographic and biological characteristics of the population according to 30-day mortality

Patients characteristics	Dead (*n* = 10)	Alive (*n* = 154)	Total	*p* value
**Age**,** average (+/-SD)**	87.4 (+/-6.5)	84.0 (+/-7.7))	84.2 (+/-7.6)	**n.s.**
**Sex**,** female**,** n (%)**	3 (30%)	71 (43.3%)	74 (45.1%)	**n.s.**
**BMI (kg/m**^**2**^**)**,** average (+/-SD)**	22.4 (4.3)	25.2 (5.3)	25.1 (5.3)	**n.s.**
**suPAR (ng/mL)**,** average +/-(SD)**	10.1(+/-4.7)	5.9 (+/-2.2)	6.2 (+/-2.6)	**0.001**
**CURB65**	3.2(+/-0.8)	2.4(+/-0.9)	2.4 (+/-0.9)	**0.006**
**PSI**	127.4 (+/-30.4)	106 (+/-25.9)	107.3 (26.6)	**0.026**
**CRP (mg/L)**,** average (+/-SD)**	145.6 (+/-107.8)	119.4 (+/-94.6)	120.9 (+/-95.3)	**n.s.**
**PCT (g/L)**,** average (+/-SD)**	5.3 (+/-7.7)	2.3 (+/-7.6)	2.5 (+/-7.6)	**0.017**
**WBC (× 10**^**9**^**/L)**,** average (+/-SD)**	13.4 (+/-10.3)	11.5 (+/-4.6)	11.7 (+/-5.1)	**n.s.**
**Urea (mmol/L)**	16.16 (+/-8.96)	9.3 (+/-5.12)	9.7 (+/-5.6)	**0.004**
**Albumin (g/L)**	30.2 (+/-3.5)	35.2 (+/-5.51)	34.9 (+/-5.53)	**0.003**
**NT-proBNP (pg/mL)**,** average (+/-SD)**	3183 (+/-1618.3)	2785 (+/-2803.7)	2799.5 (+/-2767.7)	**n.s.**

For continuous variables, the average (+**/-**SD) is indicated. For categorical variables, the number (%) is indicated

BMI: body mass index, PSI: pneumonia severity index. Median suPAR, CRP, PCT and WBC concentrations at hospital admission according to 30-day mortality CRP: C-reactive protein, PCT: procalcitonin, WBC: white blood cell

Table 2 displays the results of AUC analyses conducted on four inflammation biomarkers (suPAR, CRP, PCT and WBC) and four relevant clinical parameters usually associated with mortality (age, BMI, CURB-65, and PSI) to assess their ability to accurately differentiate between patients who died or did not die within the first month. The highest accuracy in distinguishing between the two groups was achieved by suPAR, which demonstrated an SP value of 40.3% (95% CI, 31.8–48.1) with an SE value of 100% (95% CI, 100–100) for a cut-off of 5.1 ng/mL. The clinical parameter that exhibited the greatest effectiveness was BMI, with SP and SE values of 22.5% (95% CI, 15.9–29.1) and 100% (95% CI, 100–100), respectively (Table 2).

**Table 2:** The ability of biomarkers (suPAR, CRP, PCT, WBC) and clinical parameters (age, CURB-65, PSI, BMI) to assess 30-day mortality

Variable	AUC (95% CI)	SP (95% CI)	SE (95% CI)	Cut-off
**suPAR**	80.36 (65.1–93.9)	40.3 (31.8–48.1)	100 (100–100)	5.1 ng/ml
PCT	72.5 (53.3–89.4)	14.9 (9.7–20.8)	100 (100–100)	0.1 g/l
BMI	64.11 (46.3–81.5)	22.5 (15.9–29.1)	100 (100–100)	29.1 kg/m^2^
Age	65.3 (46.9–80.2)	4.5 (1.3–8.4)	100 (100–100)	70.0 y.o.
CRP	56.1 (31.8–77.6)	0	100 (100–100)	-Inf
WBC	50.8 (31.8–69.4)	0	100 (100–100)	-Inf
CURB-65	74.5 (60.1–87.6)	18.2 (12.3–24)	100 (100–100)	1.5
PSI	71.1 (51.4–88.3)	9.7 (5.2–14.9)	100 (100–100)	79.5

The threshold value, SP and SE correspond to SEs set at 95–100%. BMI: body mass index, CRP: C-reactive protein, PCT: procalcitonin, PSI: pneumonia severity index, WBC: white blood cell

**Fig. 2:**
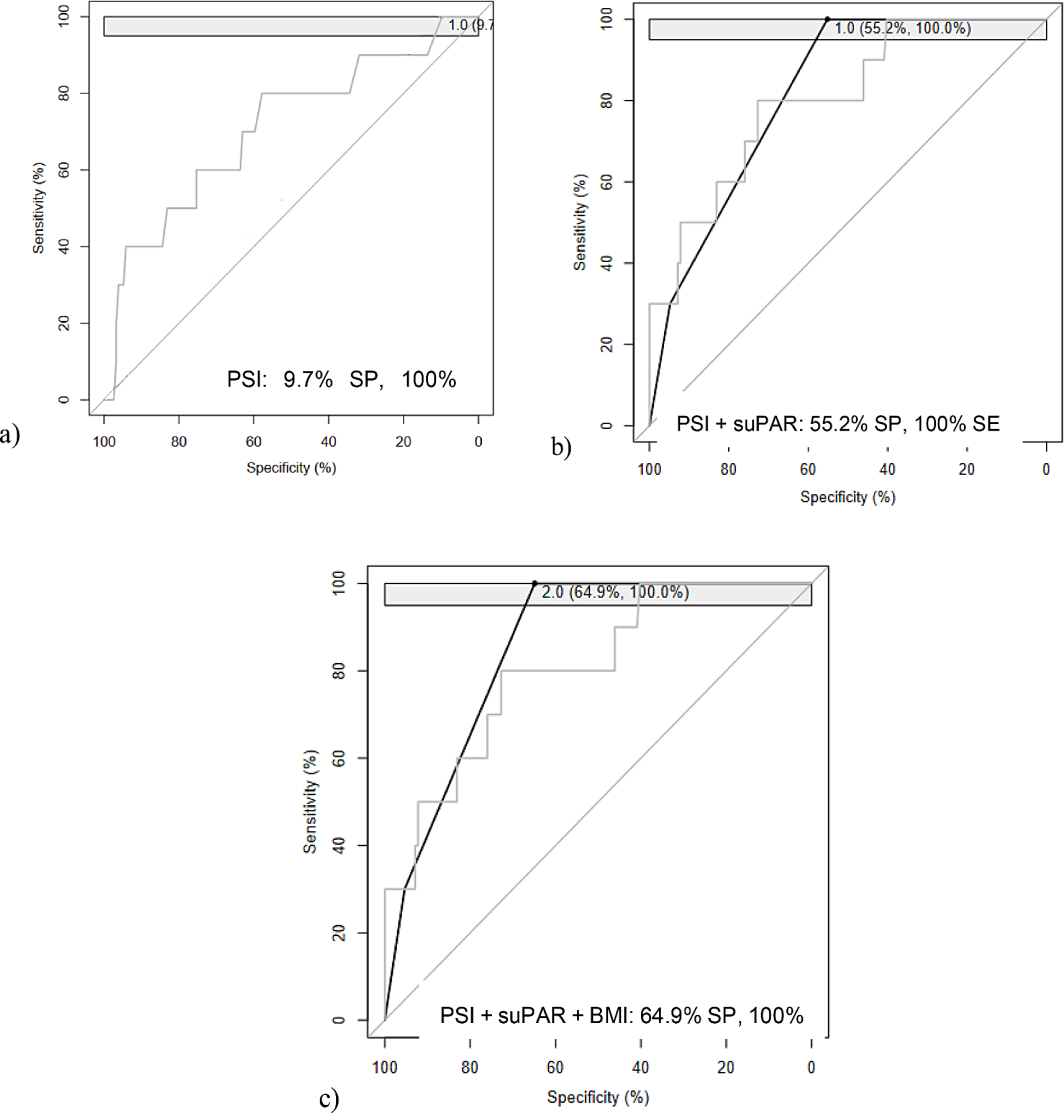
ROC curve(s) of the panel(s) (black) and of the best individual parameter (gray): **a**): PSI (Pneumonia Severity Index), **b**): PSI + suPAR panel, **c**): PSI + suPAR + BMI (Body Mass Index) panel

When the most accurate biomarker (suPAR, Fig. 2a) was merged with the most commonly used severity score (PSI), we found that compared with the use of PSI alone, the use of suPAR improved the prediction accuracy, with an SP value of 55% and an SE value of 100% (Fig. 2b). Subsequently, we evaluated the addition of a nutritional parameter, BMI, to the previous panel. This parameter was chosen for its simplicity and has been identified as a prognostic factor for pneumonia in adults [17–19]. The three-parameter panel comprising the suPAR BMI and PSI was the most accurate, with the following cut-off values: suPAR > 9.8 ng/mL, BMI < 29.3 kg/m2 and PSI > 106.5. The panel exhibited an SE of 100% (95% CI, 100–100) for an SP value of 64.9% (95% CI, 57.6–72.2) (Fig. 2c). This resulted in a significant increase in the SP when compared to the best single biomarker (suPAR) and with the best clinical parameter (BMI), providing a significant improvement (*p* = 0.03) in the capacity to assess 30-day mortality.

## Discussion

In this cohort study including 164 patients > 65 years with a suspicion of pneumonia, we found that a suPAR concentration > 5.1 ng/mL was predictive of 30-day mortality, with a sensitivity of 100% and a specificity of 40.4%. The best combination of clinical parameters and biomarkers was achieved with suPAR > 9.8 ng/mL, BMI < 29.3 kg/m2 and PSI > 106.5, which exhibited an SE of 100% (95% CI, 100–100) for an SP value of 64.9% (95% CI, 57.6–72.2) when at least two of them were above or below these cut-off values.

SuPAR is the soluble form of the cell membrane protein urokinase-type plasminogen activator receptor present on various immune and nonimmune cells and is released by the cleavage of this membrane protein, leading to a plasma concentration representative of the level of immune activation during bacterial or viral infections, cancer or autoimmune disease [20]. This biomarker has stable circadian plasma concentrations in both healthy and ill individuals, remains stable in collected plasma samples and is unaffected by the freezing and thawing process, rendering its laboratory analysis simpler [20]. Previous studies have shown promising results using suPAR for the prognosis of patients admitted to the ED, although the results are sometimes conflicting.

Holstein et al. reported that suPAR among patients consulting the ED for any cause was independently associated with 30-day mortality and with other risk assessment tools [21]. In this study, suPAR levels were greater among elderly patients (> 75 years old) than among younger patients (5.4 ng/mL vs. 3.7 ng/mL, respectively), possibly due to renal impairment among elderly individuals. Hence, the authors proposed using a cut-off value of 4 ng/mL for all ED patients vs. 5 ng/mL for elderly patients. This cut-off is consistent with our study, in which the median age was 83 years.

Similarly, Schultz et al. showed that the addition of suPAR to triage improved the identification of patients at high and low risk of 7-day mortality after their arrival at the ED, with a cut-off > 5.9 ng/mL [22].

In contrast, Jouis et al. used the suPAR alone or combined with CRP and/or lactate measurements to predict ED discharge or hospitalization in patients with nonspecific complaints and normal vital signs in a cohort with a median age of 81 years, but the suPAR was not able to predict patient outcomes [23].

In a prospective multicentre cohort of patients with community-acquired pneumonia (CAP), Luo et al. [24] reported that suPAR levels increased, especially in patients with severe CAP, with an AUC for predicting 30-day mortality of 0.77 and a threshold value of ≥ 10.2 ng/mL; however, these patients were younger than those in our cohort (mean age 50 years in patients with nonsevere pneumonia and 56 years in patients with severe pneumonia).

Song et al. [13] reported in another prospective, observational study including patients ≥ 65 years old (median age 70 years) with CAP that high suPAR levels were associated with 30-day mortality and were significantly increased in patients with severe CAP. Moreover, a positive correlation was found between suPAR levels and CURB-65 and PSI scores. Regression analysis revealed that suPAR (> 8.9 ng/mL) was an independent factor for 30-day mortality, which is a slightly greater cut-off than our study.

The usefulness of SuPAR was also test among patient with Covid-19 pneumonia. Enocsson et al. reported that suPAR was as an independent predictor of disease severity among 60 patients with Covid-19 pneumonia [25]. Giamarellos-Bourboulis et al. tested the use of suPAR for prognostication of pneumonia. In this study, suPAR concentration of ≥ 6 ng/mL has been identified as predictive of progression to SRF or death in patients hospitalized with COVID-19 pneumonia [26].

In the context of sepsis, the use of suPAR combined with a clinical score to improve prognosis was proposed by Liu et al. They showed that acute physiology and chronic health evaluation II (APACHE II) ≥ 15 ng/mL and suPAR ≥ 10.8 ng/mL were independently associated with unfavourable outcomes in patients with severe sepsis, with a mean age of 69 years [27]. The Apache II score estimates ICU mortality based on several laboratory values and patient signs, taking both acute and chronic disease into account. More recently, the SUPERIOR trial used suPAR in conbination with the qSOFA to improve its prognostic performance for sepsis detection and help for early antibiotics administration decision. A suPAR > 12ng/ml was associated with a worse outcome among patients with suspicion of sepsis [28].

Combination of clinical score and biomarker were previously reported in pneumonia [29]. Studies on the association between clinical severity scores for pneumonia, such as CURB65 and PSI, with biomarkers like CRP (C-reactive protein), procalcitonin (PCT), and suPAR (soluble urokinase plasminogen activator receptor) show varied results.

A Study demonstrated that combining PCT with severity scores (such as SOFA, CURB65, and PSI) improved the prediction of 28-day mortality, with the PCT + SOFA combination having the highest predictive value [30]. A meta-analysis compared these scores and found them reliable for evaluating 30-day mortality in cases of community-acquired pneumonia [31]. However, it was noted that these scores might be less accurate in predicting the need for ICU admission compared to other scores like SOFA [30].

In our study, to improve the ability of suPAR to predict pneumonia in individuals, we used PanelomiX, which serves as a versatile tool for constructing biomarker panels and exploring predictive models across diverse clinical scenarios. Notably, the use of PanelomiX for early infection detection in various patient cohorts has consistently achieved success [32, 33]. Specifically, when the suPAR was combined with BMI and the PSI, the cut-off value of the suPAR increased from 5.1 to 9.8 ng/mL, with a high level of sensitivity but a better specificity.

Interestingly, our results were consistent with the literature and showed that the suPAR seemed more accurate for prognostic purposes than other routinely used biomarkers, such as CRP and PCT. Previous studies reported that a weak nutritional status among elderly patients was a poor prognostic factor, specifically a low BMI, which was a predictive factor of mortality, especially in the context of an infection [34]. In this cohort, BMI seemed to be associated with 30-day mortality, but this association was not statistically significant. However, the combination of suPAR dosage > 9.8 ng/mL, BMI < 29.3 and PSI > 106.5, which is relevant in clinical practice, improved the specificity to 63%, with a sensitivity of 100%. This increase in specificity could help clinicians better triage older patients with pneumonia. This original holistic approach should be confirmed prospectively but seems promising given the interest of multimodal approaches in the elderly.

Our results are promising for several reasons. First, older patients are more at risk of developing pneumonia and have worse outcomes than younger adults are, and the use of new biomarkers to improve the accuracy of prognostication could be helpful [35, 36]. Unfortunately, most studies excluded this vulnerable population because of multiple comorbidities and inherent confounding factors. Our study specifically addresses this lack of inclusion by analysing data from a geriatric population with a clinical suspicion of pneumonia. Second, the use of readily available patient data and clinical and biological parameters such as BMI and the PSI combined with a single biomarker makes this evaluation quick and practical. Other severity scores, such as the CURB-65 score, could be an alternative and require further investigation. Third, suPAR can be administered to standard blood tube samples without specific storage or transport requirements and is performed as quickly as routine blood work. Finally, the suPAR has already been identified as a promising marker for the prognostic evaluation of multiple types of respiratory and nonrespiratory infections [8, 9, 12, 15]. Our study confirms these findings in the geriatric population.

Conversely, we identified some limitations of our study. First, the small sample size and the monocentric nature of the study limit the validity of the statistical analysis and the reproducibility of the results. Furthermore, patients admitted to ICU were excluded.

Second, suPAR is a newly identified biomarker (in contrast to classical CRP/PCT), but few laboratories perform this test routinely. This might limit the applicability of its use in smaller hospitals and outside the university setting. Third, the recruitment of patients in a tertiary university hospital may not reflect the general geriatric patient population presenting with a suspicion of pneumonia. Our study was not designed to distinguish the outcomes of bacterial and viral infections and was performed before the era of COVID-19. Further studies could explore this new aspect, as SARS-CoV-2 continues to be a source of pneumonia and mortality in the geriatric population. Finally, the kinetics of suPAR plasma concentrations are not well known. Patients’ comorbidities, previous treatments and time since the onset of pneumonia might influence the diagnostic cut-off values for this novel biomarker.

## Conclusions

We showed that a suPAR concentration > 5.1 ng/mL was predictive of 30-day mortality with a sensitivity of 100% and a specificity of 40.4% in a cohort of elderly patients suspected of having pneumonia. According to our data, when the highest level of sensitivity (100%) was needed, the best panel combination for predicting 30-day mortality was the PSI > 106.5, suPAR > 9.8 ng/mL and BMI < 29.3Kg/m2. The use of suPAR in geriatric populations presenting with suspected or diagnosed pneumonia in the ER, in conjunction with the PSI and BMI can increase the sensitivity and specificity of identifying at-risk patients for respiratory complications and mortality. Prospective studies including larger populations using this approach are needed to confirm our findings.

### Electronic supplementary material

Below is the link to the electronic supplementary material.

Supplementary Material 1

## Data Availability

The datasets used and/or analysed during the current study available from the corresponding author on reasonable request.
