# Supplementary material for: Improving prognostication of pneumonia among elderly patients: usefulness of suPAR
Source: BMC Geriatr. 2024 Aug 24;24:709. doi: 10.1186/s12877-024-05270-0 (PMC11344914; doi:10.1186/s12877-024-05270-0)
Supplement: Supplementary file 1 — Supplementary Material 1 [file 12877_2024_5270_MOESM1_ESM.docx]

**Appendix**

| **Table 1’. Demographic and clinical characteristics of the 200 patients included in the PneumO-LD-CT study** at baseline | | | | | |
| --- | --- | --- | --- | --- | --- |
|  |  |  |  |  |  |
| **Characteristics** | | **n = 200 *unless stated*** | **No. (%) or Median (IQR)** |  |  |
| Age, years (median, IQR) | |  | 84.0 (78.6–90.2) |  |  |
| ≥85 years old | |  | 93 (46.5%) |  |  |
| Female | |  | 98 (49.0%) |  |  |
| Nursing home residents | |  | 28 (14.0%) |  |  |
| Body Mass Index in kg/m^2^ | |  | 24.5 (21.5–28.6) |  |  |
| Mini Mental State Examination score | | n=162 | 24 (19–27) |  |  |
| Mini Nutritional Assessment | | n=178 | 8 (6–11) |  |  |
| Functional Independence Measure score | | n=171 | 69 (50–97) |  |  |
| Influenza vaccination within past year | | n=182 | 103 (56.6%) |  |  |
| Pneumococcal vaccination within past 5 years | | n=177 | 7 (4.0%) |  |  |
| Hospitalisation during the past 6 months | |  | 70 (35%) |  |  |
| Chronic cardiac disease | |  | 103 (51.5%) |  |  |
| Chronic Obstructive Pulmonary Disease | |  | 35 (17.5%) |  |  |
| Kidney disease |  | n=199 | 60 (30.2%) |  |  |
| Liver disease | |  | 11 (5.5%) |  |  |
| Neoplasia | |  | 17 (8.5%) |  |  |
| Smoking (past and present) | |  | 100 (50.0%) |  |  |
| History of stroke | |  | 33 (16.5%) |  |  |
| Cognitive disorders | |  | 66 (33.0%) |  |  |
| Swallowing disorders | |  | 28 (14.0%) |  |  |
| Poor oral hygiene | |  | 38 (19.0%) |  |  |
| Immunosuppressive treatment | | n=199 | 15 (7.5%) |  |  |
| Type of pneumonia | |  |  |  |  |
|  | *Community-Acquired Pneumonia* |  | *162 (81.0%)* |  |  |
|  | *Nursing Home-Acquired Pneumonia* |  | *22 (11.0%)* |  |  |
|  | *Hospital-Acquired Pneumonia (>72 h after hospitalisation)* |  | *16 (8.0%)* |  |  |
| Bronchoaspiration | | n=161 | 12 (7.5%) |  |  |
| Temperature ≥38.0°C |  |  | 116 (58.0%) |  |  |
| Cough | |  | 170 (85.0%) |  |  |
| Dyspnoea | |  | 145 (72.5%) |  |  |
| Sputum production | |  | 74 (37.0%) |  |  |
| Chest pain | |  | 35 (17.5%) |  |  |
| Crackles | |  | 171 (85.5%) |  |  |
| Decrease in respiratory sounds |  |  | 51 (25.5%) |  |  |
| Peripheral Oxygen Saturation, SpO2 <90% on admission | |  | 102 (51.0%) |  |  |
| Respiratory rate >20/min on admission |  |  | 143 (71.5%) |  |  |
| Delirium | |  | 92 (46.0%) |  |  |
| Fall | |  | 71 (35.5%) |  |  |
| CURB65 score | |  |  |  |  |
|  | *1* |  | *36 (18.0%)* |  |  |
|  | *2* |  | *75 (37.5%)* |  |  |
|  | *3* |  | *68 (34.0%)* |  |  |
|  | *4* |  | *21 (10.5%)* |  |  |
| Fine score | |  | 102 (89–123) |  |  |
|  |  |  |  |  |  |
